# Phylogenomics of Haloarchaea: The Controversy of the Genera *Natrinema-Haloterrigena*

**DOI:** 10.3389/fmicb.2021.740909

**Published:** 2021-10-07

**Authors:** Rafael R. de la Haba, Hiroaki Minegishi, Masahiro Kamekura, Yasuhiro Shimane, Antonio Ventosa

**Affiliations:** ^1^Department of Microbiology and Parasitology, Faculty of Pharmacy, University of Sevilla, Sevilla, Spain; ^2^Department of Applied Chemistry, Faculty of Science and Engineering, Toyo University, Kawagoe, Japan; ^3^Halophiles Research Institute, Chiba, Japan; ^4^Japan Agency for Marine-Earth Science and Technology, Yokosuka, Japan

**Keywords:** haloarchaea, *Halobacteria*, *Natrinema*, *Haloterrigena*, comparative genomic analysis, taxophylogenomic analysis

## Abstract

The haloarchaeal genera *Natrinema* and *Haloterrigena* were described almost simultaneously by two different research groups and some strains studied separately were described as different species of these genera. Furthermore, the description of additional species were assigned to either *Natrinema* or *Haloterrigena*, mainly on the basis of the phylogenetic comparative analysis of single genes (16S rRNA gene and more recently *rpoB’* gene), but these species were not adequately separated or assigned to the corresponding genus. Some studies suggested that the species of these two genera should be unified into a single genus, while other studies indicated that the genera should remain but some of the species should be reassigned. In this study, we have sequenced or collected the genomes of the type strains of species of *Natrinema* and *Haloterrigena* and we have carried out a comparative genomic analysis in order to clarify the controversy related to these two genera. The phylogenomic analysis based on the comparison of 525 translated single-copy orthologous genes and the Overall Genome Relatedness Indexes (i.e., AAI, POCP, ANI, and dDDH) clearly indicate that the species *Haloterrigena hispanica*, *Haloterrigena limicola*, *Haloterrigena longa*, *Haloterrigena mahii*, *Haloterrigena saccharevitans*, *Haloterrigena thermotolerans*, and *Halopiger salifodinae* should be transferred to the genus *Natrinema*, as *Natrinema hispanicum*, *Natrinema limicola*, *Natrinema longum*, *Natrinema mahii*, *Natrinema saccharevitans*, *Natrinema thermotolerans*, and *Natrinema salifodinae*, respectively. On the contrary, the species *Haloterrigena turkmenica*, *Haloterrigena salifodinae*, and *Haloterrigena salina* will remain as the only representative species of the genus *Haloterrigena*. Besides, the species *Haloterrigena daqingensis* should be reclassified as a member of the genus *Natronorubrum*, as *Natronorubrum daqingense*. At the species level, *Haloterrigena jeotgali* and *Natrinema ejinorense* should be considered as a later heterotypic synonyms of the species *Haloterrigena* (*Natrinema*) *thermotolerans* and *Haloterrigena* (*Natrinema*) *longa*, respectively. Synteny analysis and phenotypic features also supported those proposals.

## Introduction

Haloarchaea are a monophyletic group of extremely halophilic archaea affiliated to the single class *Halobacteria*, belonging to the phylum *Euryarchaeota* (Oren et al., 2017). Currently, the class *Halobacteria* comprises three orders (i.e., *Halobacteriales*, *Haloferacales*, and *Natrialbales*), six families (i.e., *Halobacteriaceae*, *Haloarculaceae*, *Halococcaceae*, *Haloferacaceae*, *Halorubraceae*, and *Natrialbaceae*), 72 genera and 289 species whose names have been validly published (Parte et al., 2020), reflecting the high diversity and complex phylogenetic relationships within the haloarchaea. In fact, recent pan-genome analysis and ancestral state reconstruction has brought to light the heterogeneity of this class, which possesses an open pan-genome, and the occurrence of genome expansion and horizontal gene transfer during the evolution of *Halobacteria* (Gaba et al., 2020).

The genera *Natrinema* and *Haloterrigena* are members of the family *Natrialbaceae*. The genus *Natrinema* was described in October 1998 (McGenity et al., 1998), just 3 months earlier than the genus *Haloterrigena* (Ventosa et al., 1999). For that reason, the latter article did not include the recently described strains of *Natrinema* for comparative purposes since the manuscript was submitted for peer-review before the acceptance of the former. Therefore, Ventosa et al. (1999), honestly according to their results, proposed the creation of the new genus *Haloterrigena* with the new species *Htg. turkmenica*, instead of a novel species within the genus *Natrinema*, which would have been more advisable. Since then, several new species affiliated to both genera have been described and, nowadays, the genus *Natrinema* comprises eight validly published species names (Minegishi and Kamekura, 2019b) while *Haloterrigena* harbors 11 species (Chen et al., 2019; Minegishi and Kamekura, 2019a). In addition, other non-validated species names have been proposed, specifically, “*Natrinema ajinwuensis*” (Mahansaria et al., 2018) and “*Natrinema thermophila*” (Kim et al., 2018), as well as isolates not-yet assigned to any existent species (*Natrinema* sp. J7-1, *Natrinema* sp. J7-2, *Haloterrigena* sp. GSL-11, and *Haloterrigena* sp. SGH1) (Post and Al-Harjan, 1988; Zhang et al., 2012; Flores et al., 2020).

Several studies have pointed out the taxonomic problems arising in the genera *Natrinema* and *Haloterrigena* from the fact that molecular markers (i.e., 16S rRNA, *atpB*, *EF-2*, *radA*, *rpoB’*, and *secY* gene sequences) and DNA–DNA hybridization data suggest an overlapping among members of both genera (Oren and Ventosa, 2002; Tindall, 2003; Wright, 2006; Enache et al., 2007; Minegishi et al., 2010; Papke et al., 2011). However, a detailed phylogenomic and comparative genomic study based on whole genome sequences has not been accomplished yet, nor was a formal proposal made to unravel the controversy between the cluster *Natrinema*/*Haloterrigena*. Moreover, the taxonomic status of the closely related genus *Natronorubrum* deserves special attention because 16S rRNA gene phylogenetic reconstructions suggest that the species *Natronorubrum sediminis* might belong to the *Natrinema*/*Haloterrigena* group, as the closest relative to *Haloterrigena daqingensis* (Ruiz-Romero et al., 2013). Since *Natronorubrum sediminis* (Gutiérrez et al., 2010) and *Haloterrigena daqingensis* (Wang et al., 2010) were proposed at almost the same time (only a 2-month gap), their close relationship was not noticed at that time. Additionally, the species *Halopiger salifodinae* seems to be properly affiliated to the genus *Halopiger* according to the 16S rRNA gene-based phylogeny, but complete *rpoB’* gene sequence analysis (which has been demonstrated to be a more advantageous phylogenetic marker than the 16S rRNA gene in the class *Halobacteria*) (Minegishi et al., 2010), indicated its closest relationship with the *Natrinema*/*Haloterrigena* cluster (Minegishi et al., 2016).

In the post-genomic era, it is possible to take advantage of big genome databases and low-cost sequencing to infer phylogenetic relationships among prokaryotes using the core orthologous genes detected in the genomes under study in order to accurately elucidate their evolutionary history (de la Haba et al., 2019). Besides, comparative genomics and Overall Genome Related Indexes (OGRI) have been proposed as approaches to inspect the evolutionary distance among species and to delineate prokaryotic taxa at family, genus and species level (Borriss et al., 2011; Chun and Rainey, 2014; Konstantinidis et al., 2017; Ramírez-Durán et al., 2021) and current taxonomy should benefit from them.

Aimed to resolve the taxonomic issues in the cluster *Natrinema*/*Haloterrigena* and related taxa within the family *Natrialbaceae*, we conducted phylogenomic and comparative genomic analyses using available dataset from public databanks. Additionally, we also obtained the whole genome sequence of a relevant type strain of this family which was missing in data banks. Several taxonomic changes are formally proposed in view of our results.

## Materials and Methods

### Genome Retrieval and Sequencing

All genome sequences from type strains of species of the family *Natrialbaceae* available until May 31st, 2020 in NCBI GenBank database were retrieved. Other additional genomes from reference (non-type) strains of *Natrinema*/*Haloterrigena* genera were also recovered (Table 1). Whole genome sequences were annotated following the NCBI Prokaryotic Genome Annotation Pipeline (PGAP) (Haft et al., 2018) to predict protein-coding genes as well as other functional genome units, such as structural RNAs and tRNAs.

**TABLE 1:** Main features of genome sequences of strains of the family *Natrialbaceae* used in this study.

Strain	Accession no.	Assembly	Level	Size (Mb)	GC%	Scaffolds	Contigs	CDS	N50	L50
*Halobiforma haloterrestris* DSM 13078^T^	FOKW00000000.1	GCA_900112205.1	Scaffold	4.50	65.4	31	32	4273	375,716	4
*Halobiforma lacisalsi* AJ5^T^	CP019285.1	GCA_000226975.3	Complete	4.38	65.2	3	3	4177	4,161,587	1
*Halobiforma nitratireducens* JCM 10879^T^	AOMA00000000.1	GCA_000337895.1	Contig	3.69	63.7	205	205	3552	47,406	25
*Halopiger aswanensis* DSM 13151^T^	RAPO00000000.1	GCA_003610195.1	Scaffold	4.87	64.4	17	18	4589	1,426,401	2
*Halopiger djelfimassiliensis* IIH2^T^	CBMA00000000.1	GCA_000455365.1	Scaffold	3.78	64.2	6	55	3671	1,082,527	2
*Halopiger goleimassiliensis* IIH3^T^	CBMB00000000.1	GCA_000455345.1	Scaffold	3.91	66.1	3	11	3756	3,025,424	1
*Halopiger salifodinae* CGMCC 1.12284^T^	FOIS00000000.1	GCA_900110455.1	Scaffold	4.27	65.4	8	9	4010	878,349	3
*Halopiger xanaduensis* SH-6^T^	NC_015666.1	GCA_000217715.1	Complete	4.36	65.2	4	4	4178	3,668,009	1
*Halostagnicola kamekurae* DSM 22427^T^	FOZS00000000.1	GCA_900116205.1	Contig	4.11	61.5	16	16	4042	1,202,185	2
*Halostagnicola larsenii* XH-48^T^	CP007055.1	GCA_000517625.1	Complete	4.13	60.9	5	5	3966	2,789,326	1
*Haloterrigena daqingensis* CGMCC 1.8909^T^	FTNP00000000.1	GCA_900156445.1	Contig	3.83	61.4	14	14	3687	859,600	2
*Haloterrigena daqingensis* JX313^T^	CP019327.1	GCA_001971705.1	Complete	3.84	61.3	4	4	3692	3,397,437	1
*Haloterrigena hispanica* CDM_1	FMZP00000000.1	GCA_900101245.1	Scaffold	3.91	61.0	135	139	3983	148,801	9
*Haloterrigena hispanica* CDM_6	FOIC00000000.1	GCA_900111485.1	Scaffold	3.96	61.0	92	100	3989	128,565	9
*Haloterrigena hispanica* DSM 18328^T^	SHMP00000000.1	GCA_004217335.1	Contig	4.26	60.7	11	11	4121	1,073,359	2
*Haloterrigena jeotgali* A29^T^	CP031303.1 (chromosome), CP031298.1, CP031299.1, CP031300.1, CP031301.1, CP031302.1, CP031304.1 (plasmids)	GCA_004799625.1	Complete	4.90	65.0	7	7	4967	3,644,881	1
*Haloterrigena limicola* JCM 13563^T^	AOIT00000000.1	GCA_000337475.1	Contig	3.52	61.8	94	94	3512	116,493	9
*Haloterrigena longa* JCM 13563^T^	JAHUQE000000000.1	GCA_020105915.1	Scaffold	4.13	63.8	6	15	4069	3,590,587	1
*Haloterrigena mahii* H13^T^	JHUT00000000.2	GCA_000690595.2	Scaffold	3.79	65.1	24	29	3707	248,588	4
*Haloterrigena saccharevitans* AB14^T^	LWLN00000000.1	GCA_001953745.1	Contig	3.98	65.3	3	3	3921	3,473,758	1
*Haloterrigena salifodinae* ZY19^T^	RQWN00000000.1	GCA_003977755.1	Scaffold	4.96	64.5	11	14	4761	1,204,032	2
*Haloterrigena salina* JCM 13891^T^	AOIS00000000.1	GCA_000337495.1	Contig	4.84	65.2	71	71	4540	151,334	11
*Haloterrigena* sp. H1	SMZK00000000.1	GCA_005938085.1	Contig	4.26	61.5	9	9	4253	3,035,199	1
*Haloterrigena thermotolerans* DSM 11552^T^	AOIR00000000.1	GCA_000337115.1	Contig	3.90	65.4	68	68	3862	162,183	9
*Haloterrigena turkmenica* DSM 5511^T^	NC_013743.1	GCA_000025325.1	Complete	5.44	64.2	7	7	5167	3,889,038	1
*Haloterrigena turkmenica* WANU15	LKCV00000000.1	GCA_001483125.1	Contig	2.95	64.0	574	574	3202	15,902	50
*Halovivax asiaticu*s JCM 14624^T^	AOIQ00000000.1	GCA_000337515.1	Contig	3.24	64.5	24	24	3115	327,817	4
*Halovivax ruber* XH-70^T^	NC_019964.1	GCA_000328525.1	Complete	3.23	64.3	1	1	3099	3,223,876	1
*Natrarchaeobaculum aegyptiacum* JW/NM-HA 15^T^	CP019893.1	GCA_002156705.1	Complete	3.93	64.1	1	1	3745	3,930,546	1
*Natrarchaeobaculum sulfurireducens* AArc1^T^	CP024047.1	GCA_003430825.1	Complete	3.79	62.4	3	3	3576	3,521,804	1
*Natrarchaeobius chitinivorans* AArcht4^T^	REGA00000000.1	GCA_003841505.1	Contig	4.57	61.9	48	48	4382	170,161	8
*Natrarchaeobius halalkaliphilus* AArcht-Sl^T^	REFY00000000.1	GCA_003841485.1	Contig	3.51	61.1	12	12	3409	639,802	3
*Natrialba aegyptia* DSM 13077^T^	AOIP00000000.1	GCA_000337535.1	Contig	4.62	62.0	66	66	4429	145,225	7
*Natrialba asiatica* DSM 12278^T^	AOIO00000000.1	GCA_000337555.1	Contig	4.40	62.4	49	49	4188	174,934	7
*Natrialba chahannaoensis* JCM 10990^T^	AOIN00000000.1	GCA_000337135.1	Contig	4.31	60.4	106	106	4030	129,612	13
*Natrialba hulunbeirensis* JCM 10989^T^	AOIM00000000.1	GCA_000337575.1	Contig	4.16	61.7	48	48	3834	159,578	8
*Natrialba magadii* ATCC 43099^T^	NC_013922.1	GCA_000025625.1	Complete	4.44	61.0	4	4	4154	3,751,858	1
*Natrialba swarupiae* ESP3B_9^T^	VTAW00000000.1	GCA_008245225.1	Contig	4.20	62.5	99	99	3969	129,190	11
*Natrialba taiwanensis* DSM 12281^T^	AOIL00000000.1	GCA_000337595.1	Contig	4.64	61.5	70	70	4399	199,614	9
*Natrinema altunense* AJ2^T^	JNCS00000000.1	GCA_000731985.1	Contig	3.77	64.6	20	20	3688	425,349	4
*Natrinema altunense* 1A4-DGR	JXAN00000000.1	GCA_000815265.1	Contig	3.72	64.8	215	215	5159	33,862	34
*Natrinema altunense* 4.1R	SHMR00000000.1	GCA_004209855.1	Scaffold	3.67	64.9	12	81	3631	1,929,556	1
*Natrinema altunense* JCM 12890^T^	AOIK00000000.1	GCA_000337155.1	Contig	3.77	64.5	52	52	3698	184,807	7
*Natrinema ejinorense* JCM 13890^T^	NXNI00000000.1	GCA_002494345.1	Contig	4.48	63.9	3	3	4337	3,988,345	1
*Natrinema gari* JCM 14663^T^	AOIJ00000000.1	GCA_000337175.1	Contig	4.02	63.7	88	88	3997	126,340	11
*Natrinema pallidum* BOL6-1	CP040637.1	GCA_005890195.1	Complete	3.78	64.3	3	3	3723	3,503,953	1
*Natrinema pallidum* DSM 3751^T^	AOII00000000.1	GCA_000337615.1	Contig	3.92	63.7	115	115	3852	88,603	17
*Natrinema pellirubrum* DSM 15624^T^	NC_019962.1 (chromosome), NC_019963.1, NC_019967.1 (plasmids)	GCA_000230735.3	Complete	4.35	64.0	3	3	4249	3,790,479	1
*Natrinema salaciae* DSM 25055^T^	FOFD00000000.1	GCA_900110865.1	Scaffold	4.86	65.0	11	15	4634	865,606	3
*“Natrinema thermophila*” CBA1119	PDBS00000000.1	GCA_002572525.1	Contig	5.06	62.3	9	9	4965	4,087,412	1
*Natrinema* sp. J7-1	AJVG00000000.1	GCA_000493245.1	Contig	3.67	64.4	42	42	3632	196,646	6
*Natrinema* sp. J7-2	NC_018224.1	GCA_000281695.1	Complete	3.79	64.1	2	2	3706	3,697,626	1
*Natrinema versiforme* BOL5-4	CP040330.1	GCA_005576615.1	Complete	4.67	63.4	5	5	4514	3,747,116	1
*Natrinema versiforme* JCM 10478^T^	AOID00000000.1	GCA_000337195.1	Contig	4.19	64.0	72	72	4146	121,463	13
*Natronobacterium gregoryi* SP2^T^	NC_019792.1	GCA_000230715.3	Complete	3.79	62.2	1	1	3710	3,788,356	1
*Natronobacterium texcoconense* DSM 24767^T^	FNLC00000000.1	GCA_900104065.1	Scaffold	4.01	62.9	9	10	3976	1,245,734	2
*Natronococcus amylolyticus* DSM 10524^T^	AOIB00000000.1	GCA_000337675.1	Contig	4.42	64.4	44	44	4320	232,276	7
*Natronococcus jeotgali* DSM 18795^T^	AOIA00000000.1	GCA_000337695.1	Contig	4.50	64.4	170	170	4458	76,066	20
*Natronococcus occultus* SP4^T^	NC_019974.1	GCA_000328685.1	Complete	4.31	64.6	3	3	4174	4,013,216	1
*Natronolimnobius baerhuensis* CGMCC 1.3597^T^	MWPH00000000.1	GCA_002177135.1	Contig	3.91	60.2	8	8	3745	1,261,254	2
*Natronolimnohabitans innermongolicus* JCM 12255^T^	AOHZ00000000.1	GCA_000337215.1	Contig	4.59	64.3	121	121	4384	96,333	18
*Natronorubrum aibiense* 7-3^T^	CP045488.1	GCA_009392895.1	Complete	4.35	61.3	4	4	4130	3,352,994	1
*Natronorubrum bangense* JCM 10635^T^	AOHY00000000.1	GCA_000337715.1	Contig	4.11	60.4	62	62	3982	138,654	10
*Natronorubrum sediminis* CGMCC 1.8981^T^	FNWL00000000.1	GCA_900108095.1	Scaffold	3.78	61.1	6	9	3583	1,300,740	2
*Natronorubrum sulfidifaciens* JCM 14089^T^	AOHX00000000.1	GCA_000337735.1	Contig	3.46	61.8	63	63	3408	225,522	5
*Natronorubrum texcoconense* B4^T^	FNFE00000000.1	GCA_900100335.1	Contig	4.64	63.6	11	11	4423	457,630	3
“*Natronorubrum thiooxidans*” HArc	FTNR00000000.1	GCA_900156475.1	Scaffold	4.21	60.9	62	63	4067	250,216	6
*Natronorubrum tibetense* GA33^T^	ARPH00000000.1	GCA_000383975.1	Scaffold	4.93	62.3	5	10	4649	4,057,512	1

The genome sequence of the type strain of *Haloterrigena longa* was not available in any searched public database (NCBI GenBank, JGI Genome Portal, Global Catalog of Type Strain). Since that sequence data was quite relevant for the present work, we obtained the type material from the Japanese Collection of Microorganisms for the aforementioned strain (JCM 13563) and further processed it in order to obtain its whole genome sequence. High-quality genomic DNA was extracted using the QIAmp DNA Mini Kit (Qiagen) following the manufacturer’s instructions. Library preparation was performed using a combination of paired-end and mate pair strategies to generate short-insert and long-insert paired-end DNA libraries, respectively. DNA fragments were sequenced on an Illumina MiSeq platform to obtain 2 × 301-bp short-insert paired-end reads (SIPERs) and 2 × 301-bp long-insert paired-end reads (LIPERs). Downstream analyses were carried out as previously described (Ramírez-Durán et al., 2020). In brief, sequencing reads were quality filtered and trimmed using BBTools v.38.44 (Bushnell, 2020) and then assembled with SPAdes v.3.13.0 (Bankevich et al., 2012) using combined SIPERs and LIPERs as input. Automatic annotation of the draft genome was achieved using PGAP (Haft et al., 2018) as indicated above, that includes prediction of protein-coding genes, as well as other functional genome units such as structural RNAs, tRNAs, small RNAs and pseudogenes using a combination of *ab initio* gene prediction algorithms with homology based methods.

### Phylogenetic and Phylogenomic Treeing

The 16S rRNA gene sequences from the type strains of species of the family *Natrialbaceae* were downloaded from GenBank/EMBL/DDBJ databases or extracted from the whole genome sequences and then, aligned and used to calculate similarity matrixes and to construct neighbor-joining (NJ) (Saitou and Nei, 1987), maximum-parsimony (MP) (Fitch, 1971), and maximum-likelihood (MP) (Felsenstein, 1981) phylogenetic trees in ARB v.6.0.5 software package (Westram et al., 2011). Jukes-Cantor model of DNA evolution (Jukes and Cantor, 1969) was selected to correct the distance matrix. General Time Reversible model (Tavaré, 1986) with gamma-distribution and proportion of invariant sites to estimate rate heterogeneity over sites (GTR + Γ + I) was used to infer ML phylogeny. Branch support was assessed by 1,000 bootstrap pseudo-replicates (Felsenstein, 1985).

Since 16S rRNA gene-based phylogenies have been demonstrated not to be reliable to determine in-depth evolutionary relationships within the class *Halobacteria* and their results must be regarded with caution and carefully checked (Papke, 2009; Corral et al., 2018; de la Haba et al., 2018; Infante-Domínguez et al., 2020), a more robust and accurate phylogenomic approach was attempted. Firstly, pan- and core-genome datasets were determined using an all-vs.-all Blastp comparison among the translated CDS features of the annotated genomes under study, as previously described (de la Haba et al., 2019). Then, translated single-copy core gene sequences were individually aligned with Muscle (Edgar, 2004) and concatenated into a super-protein alignment, which was further analyzed to generate the phylogenomic tree by means of the approximately maximum-likelihood algorithm implemented in FastTreeMP v.2.1.8 (Price et al., 2010). Jones-Taylor-Thornton model of amino acid evolution (Jones et al., 1992) with a single rate for each site (JTT + CAT) was applied for phylogenomic reconstruction. Tree branch support was inferred using the Shimodaira-Hasegawa test (Shimodaira and Hasegawa, 1999).

Both, 16S rRNA gene-based and phylogenomic trees, were managed, displayed and annotated using the online tool iTOL v.5.7 (Letunic and Bork, 2021).

### Comparative Genomic Analyses

Overall Genome Relatedness Indexes (OGRI) were calculated for all-vs.-all genome pairs. Specifically, Orthologous Average Nucleotide Identity (OrthoANI) was determined using the OrthoANIu Tool (Yoon et al., 2017) which depends on USEARCH v8.1.1861, the digital DNA-DNA hybridization (dDDH) was inferred by means of the Genome-to-Genome Distance Calculator (GGDC) (formula 2) (Meier-Kolthoff et al., 2013), the Average Amino-acid Identity (AAI) was estimated using the aai.rb script from the Enveomics collection (Rodriguez-R and Konstantinidis, 2016) and, finally, the Percentage Of Conserved Proteins (POCP) was calculated with a homemade Perl script as described elsewhere (Qin et al., 2014).

Synteny analysis among selected representative genomes within the family *Natrialbales* was carried out to detect conservation of homologous genes and gene order across closed relatives. Because synteny can be affected by sequence fragmentation (Liu et al., 2018), draft genome contigs were reordered prior to infer synteny blocks using a gold standard genome (i.e., complete genome sequence) of a closely related species as a reference, using the Mauve Contig Mover functionality (Rissman et al., 2009). Conserved blocks were identified after Blastn pairwise comparisons (e-value ≤ 10^–3^) between the rearranged genomes and synteny plots were visualized using Easyfig v.2.2.3 (Sullivan et al., 2011).

## Results and Discussion

### The 16S rRNA Gene Sequence Analysis Unveils the Taxonomic Problems Arising Within the Genera *Haloterrigena* and *Natrinema*

**FIGURE 1:**
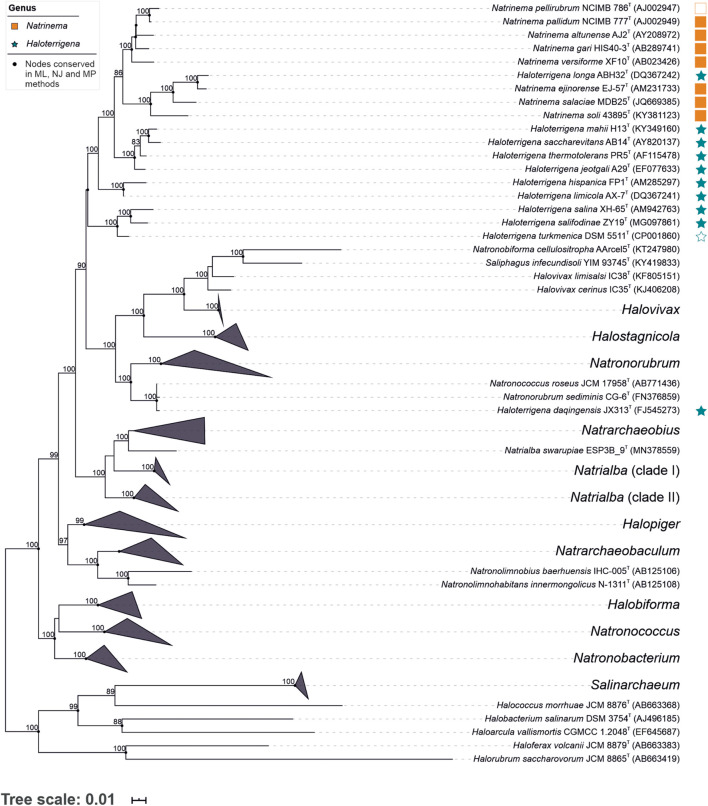
Maximum-likelihood phylogenetic tree based on the 16S rRNA gene sequence comparison of members of the genera *Natrinema* and *Haloterrigena* and representatives of the most closely related genera of the family *Natrialbaceae*. Bootstrap values ≥ 70% (based on 1,000 *pseudo*-replicates) are shown above the branches. Bar, 0.01 changes per nucleotide position. Empty square and star indicate the type species of the corresponding genus.

To gain a general overview of the current taxonomic situation of the family *Natrialbaceae* we reconstructed a phylogeny based on the 16S rRNA gene sequences (the most widely used molecular marker in modern prokaryotic systematics) including all type strains of the species with validly published names within that family (Figure 1). As hinted at previous studies (Tindall, 2003; Wright, 2006; Gupta et al., 2016), our results confirm that neither the genus *Natrinema* nor the genus *Haloterrigena* constituted monophyletic groups, but the constituent species of both genera were intermingled into a single monophyletic cluster, with the exception of the species *Haloterrigena daqingensis* which clustered together to *Natronorubrum sediminis* and *Natronococcus roseus*, distantly related to the rest of the species of *Natrinema*/*Haloterrigena*. Other problematic (polyphyletic or paraphyletic) genera within this family were *Halovivax*, *Natrialba*, *Natronococcus*, and *Natronorubrum* (Figure 1).

The 16S rRNA gene sequence similarities among the type species within the genera *Natrinema* and *Haloterrigena*, independently considered, ranged between 99.5–95.3% and 99.0–94.4%, respectively, while the sequence similarities between both genera varied from 99.0 to 94.6%, by far above the threshold value for differentiating prokaryotic genera (<94.5%) (Yarza et al., 2014). Therefore, intra- and inter-genera sequence similarities overlap almost entirely, which indicates a rather fuzzy delineation between those two genera. With regards to species delineation, the following monophyletic groups sharing equal or more than 98.65% sequence similarity (generally accepted as the prokaryotic species cutoff value) (Kim et al., 2014) could be observed: *Natrinema pellirubrum*—*Natrinema pallidum*; *Natrinema ejinorense*—*Haloterrigena longa*; *Haloterrigena mahii*—*Haloterrigena saccharevitans*—*Haloterrigena thermotolerans*; *Haloterrigena hispanica*—*Haloterrigena limicola*; *Haloterrigena daqingensis*—*Natronococcus roseus*—*Natronorubrum sediminis*. Besides, other potential species synonymy could be detected in the family *Natrialbaceae*: *Halobiforma haloterrestris*—*Halobiforma lacisalsi*; *Halopiger aswanensis*—*Halopiger thermotolerans*—*Halopiger xanaduensis*; *Halostagnicola alkaliphila*—*Halostagnicola bangensis*; *Halovivax asiaticus*—*Halovivax ruber*; and *Natrialba aegyptia*—*Natrialba taiwanensis*—*Natrialba asiatica*. Despite that different species could sometimes share values above the indicated threshold, the groups mentioned here should be carefully checked to detect the existence of synonymy.

### Taxophylogenomics and Overall Genome Related Indexes Values Prove the Proposal to Keep the Genera *Natrinema* and *Haloterrigena* as Separated Taxa

To confirm the results noted after 16S rRNA gene sequence analysis, a more robust and determinative phylogenomic analysis was carried out. For that purpose, all genome sequences from type strains of the species of the family *Natrialbaceae* as well as other non-type strains of the genera *Natrinema* and *Haloterrigena* available in NCBI GenBank database at the time of the study were recovered. Since the genome data for the type strain of *Haloterrigena longa* could not be retrieved and because this species requested special attention given its close relationship to *Natrinema ejinorense* (as indicated above), we sequenced and analyzed it. A total of ∼0.32 and ∼2.12 Gb from paired-end and mate pair libraries, respectively, were obtained after trimming and filtering. Average insert size was computationally estimated to be ∼550 bp for paired-end and ∼2,000 bp for mate pair datasets. Assembly yielded a 4.13 Mb, 6 scaffolds genome with a N50 of 3,590,587 bp and a coverage of 78X. Table 1 shows all the genome sequences used in this study, as well as their main features.

**FIGURE 2:**
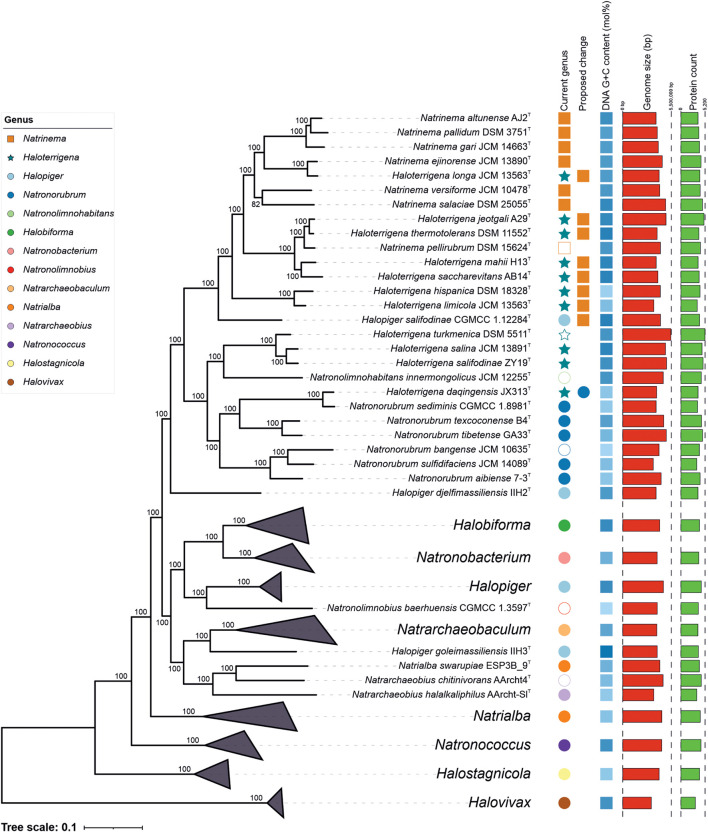
Approximate maximum-likelihood phylogenomic tree based on the concatenation of the translated sequence of the 525 single-copy genes shared by the type strains of members of the genera *Natrinema* and *Haloterrigena* and related taxa of the family *Natrialbaceae* under study. Bootstrap values ≥ 70% (based on Shimodaira-Hasegawa-like local support) are shown above the branches. Bar, 0.1 changes per nucleotide position. Empty symbols indicate the type species of the corresponding genus.

Phylogenomic trees inferred from the concatenation of the 525 amino acid sequences of the orthologous single-copy genes present in the type strain genomes (Figure 2) and in all the genomes under study (Supplementary Figure 1) were obtained. A previous study focused on the evolution of the class *Halobacteria* has also reported phylogenomic core trees that concurs with our results, although those phylogenetic reconstructions were based on only 45 orthologous core genes and did not include all the representative genomes within the *Natrialbales* (Gaba et al., 2020). As might be expected, the topology of our phylogenomic tree was not totally in agreement to the 16S rRNA gene phylogeny, but the clusters obtained were better supported in the phylogenomic tree, with 100% bootstrap in almost all bifurcations. Most significantly, the strains from the cluster *Natrinema*/*Haloterrigena* (which could not be distinguished either from each other, as in the 16S rRNA tree) did not form a monophyletic group, even when excluding the species *Haloterrigena daqingensis*. In particular, *Haloterrigena turkmenica* (the type species of the genus), *Haloterrigena salifodinae*, and *Haloterrigena salina* clustered together and separated from the other *Natrinema*/*Haloterrigena* members. Therefore, our results indicate that merging both genera is not convenient, while transferring some current *Haloterrigena* species (i.e., *Htg. hispanica*, *Htg. jeotgali*, *Htg. limicola*, *Htg. longa*, *Htg. mahii*, *Htg. saccharevitans*, *Htg. thermotolerans*) to the genus *Natrimena* seems more appropriate. That way the genus *Haloterrigena* would remain composed of the species *Htg. turkmenica*, *Htg. salina*, and *Htg. salifodinae*. Furthermore, the species *Haloterrigena daqingensis* formed a monophyletic group with all the *Natronorubrum* species, thus suggesting its reclassification as a member of the latter genus. It is worth noting that the species *Halopiger salifodinae* did not affiliate with the other *Halopiger* species but was closely related to the *Natrinema*/*Haloterrigena* group. This taxon might belong, indeed, to the latter group or, alternatively, it might constitute a new separate genus within the *Natrialbaceae*. In order to unravel this issue, an in-depth analysis of OGRI values may be determinative.

The reference non-type strains whose genome sequences were included in this study showed that all of them clustered together to their respective type strain, except for the strain *Haloterrigena turkmenica* WANU15, which might be part of the genus *Natronolimnohabitans*; however, it must be noted that the genome sequence of *Haloterrigena turkmenica* WANU15 has been confirmed to be contaminated (Lee et al., 2017) and, thus, this result must be observed with caution. Concerning the unnamed strains analyzed (i.e., *Natrinema* sp. J-1, *Natrinema* sp. J-2, and *Haloterrigena* sp. H1), the two first probably belong to the species *Natrinema gari*, whereas the latter might be regarded as a new species into the *Natrinema*/*Haloterrigena* archaeal set. Phylogenomic tree also uncover some other taxonomic problems arising within the *Natrialbaceae*, such as the polyphyly of the genera *Natrialba* and *Halopiger*, but they are out of the scope of this study.

**FIGURE 3:**
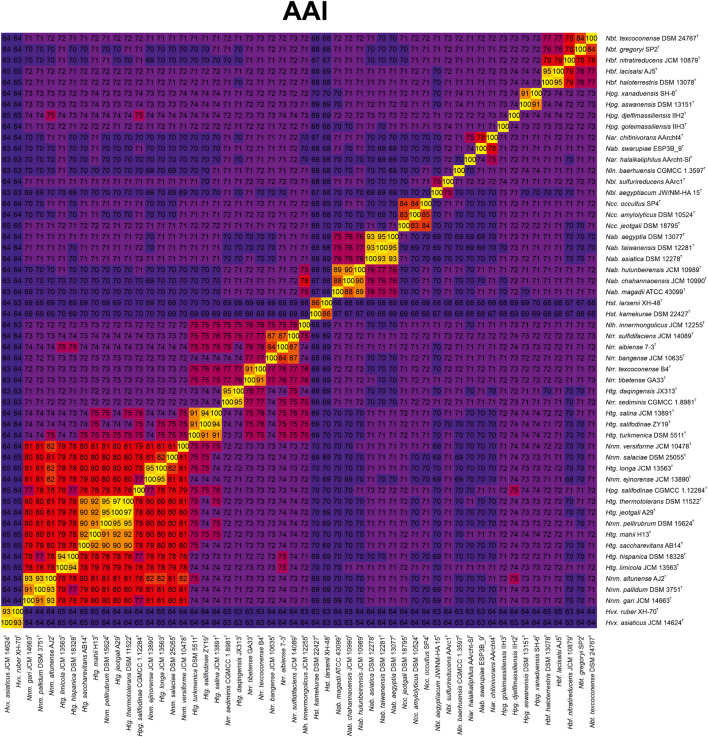
Heatmap of AAI relatedness among the type strains of members of the genera *Natrinema* and *Haloterrigen*a and representatives of the other genera of the family *Natrialbaceae*.

Aimed to shed light on the classification of the *Natrialbaceae*, several OGRI types were calculated, in particular those mostly accepted to delineate taxa at the prokaryotic genus and species level. Methods to demarcate genera have been proposed that are based on either AAI (Konstantinidis and Tiedje, 2007) or the POCP (Qin et al., 2014). The former approach sets a cutoff value for genus demarcation of 65% AAI (Konstantinidis et al., 2017); however, this threshold cannot be universally employed for all bacterial and archaeal lineages. In fact, if we would use the 65% AAI cutoff all the genera within the *Natrialbaceae*, apart from the genus *Halovivax*, should be merged in a single one since they shared AAI values equal or above 67% (Figure 3 and Supplementary Figure 2). Therefore, AAI values might be useful for genera demarcation in this family, but a different boundary needs to be established for it. Previous studies have pointed out the convenience to set lineage specific OGRI limits to define prokaryotic genera (Barco et al., 2020). Genus demarcation boundaries were determined for the family *Natrialbaceae* after detailed inspection of AAI values for all pairwise genome comparisons (Figure 3 and Supplementary Figure 2), in agreement with the phylogenomic trees (Figure 2 and Supplementary Figure 1), to avoid the existence of polyphyletic genera. Thus, we propose a cutoff value of ≤ 76% AAI to differentiate genera within the family *Natrialbaceae*, a robust and consistent threshold according to the observed evolutionary relationships among members of this family. By using this threshold, the species *Haloterrigena turkmenica*, *Haloterrigena salifodinae*, and *Haloterrigena salina* will be retained as the only members of *Haloterrigena*. Moreover, the species *Haloterrigena daqingensis* should be transferred to the genus *Natronorubrum*. Finally, the remaining species of *Haloterrigena*, the species *Halopiger salifodinae* and all the *Natrinema* species should be joined together into a single genus. Since the genus *Natrinema* has priority over the other two, all the aforementioned species should be reclassified as members of *Natrinema*. Our proposed genus limit should also have consequences in the taxonomic status of other genera of the family *Natrialbaceae*, such as the convenience to merge the genera *Halobiforma* and *Natronobacterium*, the transfer of *Natrialba swarupia* into the genus *Natrarchaeobius* and the need to revisit the affiliation of *Halopiger goleimassiliensis* and *Halopiger djelfimassiliensis* outside the genus *Halopiger*. Nevertheless, additional studies including all the type strains of the species of those genera is required, which is beyond the subject of the present article.

On the other hand, the POCP method sets a genus boundary at a value of 50% (Qin et al., 2014). Nevertheless, that limit cannot be applied to the family *Natrialbaceae* since all the constituent genera shared values above it. It has been discussed that this cutoff value was arbitrarily established (Barco et al., 2020), so, according to our results (Supplementary Figure 3) we can propose a threshold at a POCP value of < 66% for genus demarcation in this family. Nevertheless, this genomic index seems not to be as accurate as AAI and in borderline cases interpretation of results may be unclear. For example, the group formed by *Haloterrigena turkmenica*, *Haloterrigena salifodinae*, and *Haloterrigena salina* (which seemed to constitute an independent genus as explained above) could not be clearly separated from the *Haloterrigena daqingensis*/*Natronorubrum* spp. cluster, from the genus *Natronolimnohabitans*, or from the rest of the strains of the *Natrinema*/*Haloterrigena* clade using our proposed POCP-based genus cutoff. Another outlier was the low POCP values of the strain *Natrinema altunense* 1A4-DGR with respect to most of the strains within the *Natrinema*/*Haloterrigena* cluster, indicating some confidence issues for this index. Besides, other genera within the family *Natrialbaceae* that could not be distinguished using POCP index but whose unification is not supported by phylogenomic tree were *Natrarchaeobaculum*—*Natrarchaeobius*—*Natronolimnobius*; *Halobiforma*—*Halopiger*. Hence, we discourage taxonomist from using POCP method to define genera within the family *Natrialbaceae*.

**FIGURE 4:**
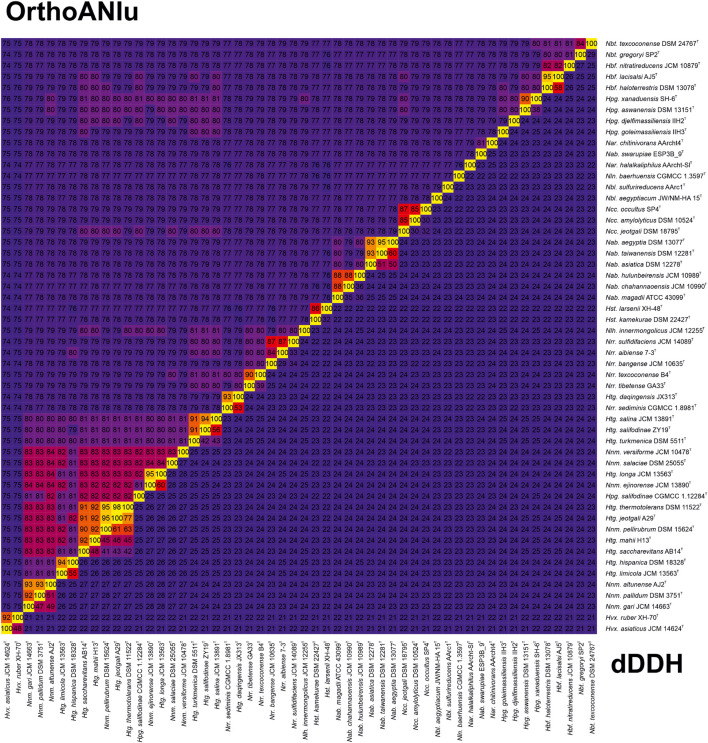
Heatmap of OrthoANIu **(upper triangle)** and dDDH **(lower triangle)** relatedness among the type strains of members of the genera *Natrinema* and *Haloterrigen*a and representatives of the other genera of the family *Natrialbaceae*.

A longer list of OGRI has been proposed to be useful for prokaryotic species delineation (Palmer et al., 2020), such as AAI (Konstantinidis and Tiedje, 2005) –which can also be employed for genus demarcation–, ANIb (Goris et al., 2007), ANIm, TETRA (Richter and Rossello-Mora, 2009), MUMi (Deloger et al., 2009), dDDH (Meier-Kolthoff et al., 2013), gANI, alignment fraction (Varghese et al., 2015), OrthoANI (Lee et al., 2016), and FastANI (Jain et al., 2018). Among them, two of the most widely used for taxonomic purposes at species level are dDDH and OrthoANI, with widely accepted cutoff values of 70% (Auch et al., 2010) and 95–96% (Goris et al., 2007; Richter and Rossello-Mora, 2009; Chun and Rainey, 2014), respectively. We calculated these two OGRI for the family *Natrialbaceae* (Figure 4 and Supplementary Figure 4) with the aim to identify the existence of synonymy between recognized species names and to properly affiliate unnamed strains to a species. A first glimpse of OrthoANI/dDDH results showed several borderline genome pairs (94% OrthoANI and ∼55% dDDH) in our dataset, in particular *Haloterrigena hispanica* DSM 18328^T^/*Haloterrigena limicola* JCM 13563^T^, *Haloterrigena daqingensis* JX313^T^/CGMCC 1.8909^T^/*Natronorubrum sediminis* CGMCC 1.8961^T^, and *Haloterrigena salifodinae* ZY19^T^/*Haloterrigena salina* JCM 13891^T^, but they cannot be regarded as synonyms because they are still below the species threshold values and might indicate a recent speciation event. Following this criterion, the non-type strains *Haloterrigena hispanica* CDM_1 and *Haloterrigena hispanica* CDM_6 seemed to be misclassified and they should be described as a separated species from *Haloterrigena hispanica*, although a further descriptive characterization is required for this purpose. Unfortunately, none of both strains are available in public microbial culture collections. Similarly, the strain *Haloterrigena* sp. H1, sharing ≤ 89% OrthoANI and ≤ 39% dDDH values with respect to any of the analyzed strains in the family *Natrialbaceae*, constitutes a novel species within the cluster *Natrinema*/*Haloterrigena*, but access to the biological resource is needed before to make any formal proposal. Our study also indicated that the species “*Natrinema thermophila*” (Kim et al., 2018) and “*Natronorubrum thiooxidans*” (Sorokin et al., 2005) (names effectively but not validly published) should be unequivocally considered as novel taxa within their respective genera, although those names need to be validated beforehand. More uncertain was the taxonomic differentiation of several genome pairs within the fuzzy zone (95% OrthoANI and 60–63% dDDH), specifically *Natrinema pellirubrum* DSM 15624^T^/*Haloterrigena jeotgali* A29^T^, *Natrinema pellirubrum* DSM 15624^T^/*Haloterrigena thermotolerans* DSM 11522^T^, and *Natrinema ejinorense* JCM 13890^T^/*Haloterrigena longa* JCM 13563^T^. Additionally, it must be noted that when using formula 1 and 3 (instead of formula 2) for dDDH calculation the results for the aforementioned genome pairs were 64–65%, 67–68%, and 70%, respectively, making more challenging their proper taxonomic classification. In those cases, the sole use of OGRI values was not discriminative enough as to make a decision on their taxonomy and additional genomic and phenotypic data must be provided. On the other hand, OrthoANI and dDDH values doubtlessly indicate that each of the following groups of strains belongs to the same species: *Natrinema gari* JCM 14663^T^/*Natrinema* sp. J7-1/*Natrinema* sp. J7-2, *Natrinema pallidum* DSM 3751^T^/*Natrinema pallidum* BOL6-1, *Natrinema altunense* JCM 12890^T^/*Natrinema altunense* AJ2^T^/*Natrinema altunense* 4.1R/*Natrinema altunense* 1A4-DGR, *Haloterrigena hispanica* CDM_1/*Haloterrigena hispanica* CDM_6, *Haloterrigena jeotgali* A29^T^/*Haloterrigena thermotolerans* DSM 11522^T^, *Haloterrigena daqingensis* JX313^T^/*Haloterrigena daqingensis* CGMCC 1.8909^T^, and *Natronolimnohabitans innermongolicus* JCM 12255^T^/*Haloterrigena turkmenica* WANU15. Therefore, the species *Haloterrigena jeotgali* should be considered as a later heterotypic synonym of *Haloterrigena thermotolerans* and the strains *Natrinema* sp. J7-1, *Natrinema* sp. J7-2, and *Haloterrigena turkmenica* WANU15 should be renamed as *Natrinema gari* J7-1, *Natrinema gari* J7-2, and *Natronolimnohabitans innermongolicus* WANU15, respectively. Other putative ambiguous synonyms were detected, such as those for the species *Natrialba aegyptia*/*Natrialba taiwanensis* and *Halobiforma haloterrestris*/*Halobiforma lacisalsi*, but the convenience to be merged or not should be accomplished in future studies.

### Synteny Analysis Applied to Elucidation of Uncertain Synonyms Into the *Natrinema*/*Haloterrigena*

The evolutionary processes that lead to diversity, chromosomal dynamics, and rearrangement rates between species can be assessed by means of the analysis of the synteny among two or more genomes, that is, the spatial distribution of locally collinear blocks (Bhutkar et al., 2006). Thus, an approach to gain insight into the evolutionary distance between two species is to inspect the synteny of the genome sequences under study (Borriss et al., 2011; Ramírez-Durán et al., 2021). As indicated in the previous section, OGRI values equal to the species cutoffs were not able to reliably solve the taxonomic status of several species and so, the synteny analysis might shed light to elucidate the affiliation of those uncertain taxa.

**FIGURE 5:**
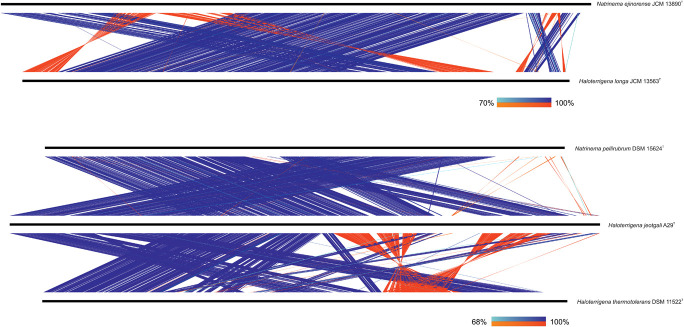
Synteny plot between the genomes of *Natrinema ejinorense* and *Haloterrigena longa* **(above)** and among the genomes of *Natrinema pellirubrum*, *Haloterrigena jeotgali*, and *Haloterrigena thermotolerans* **(below)**. Only matches with ≥ 500 bp alignment length and ≥ 90% identity are shown.

Specifically, we have evaluated, on the one hand, the synteny between *Natrinema ejinorense* JCM 13890^T^ and *Haloterrigena longa* JCM 13563^T^ and, on the other hand, the synteny among *Natrinema pellirubrum* DSM 15624^T^, *Haloterrigena jeotgali* A29^T^, and *Haloterrigena thermotolerans* DSM 11522^T^ (Figure 5). As can be observed, although some genomic rearrangements could be evidenced, all comparisons showed high levels of conservation of locally collinear blocks. It must be noted that the synteny between *Haloterrigena jeotgali* A29^T^ and *Haloterrigena thermotolerans* DSM 11522^T^ seemed to be more disorganized than that for other genome pairs; however, this fact is due to the elevated fragmentation of the genome sequence from *Haloterrigena thermotolerans* DSM 11522^T^ (68 scaffolds and a N50 of 162,183 bp), which reduces the robustness of the synteny analysis. In any case, the synteny results are not so relevant for such genome pair since OGRI values undoubtedly demonstrated the synonym between those species, as stated earlier. The other genome sequences analyzed here for synteny comparisons possessed high-quality, with a minimum N50 of 3.59 Mb and, therefore, they met the requirements to be confidently used for this purpose (Liu et al., 2018).

Our results concerning the study of regions of local collinearity support the union of *Natrinema ejinorense* and *Haloterrigena longa* and of *Natrinema pellirubrum* and *Haloterrigena jeotgali*/*Haloterrigena thermotolerans* as a single species, respectively. Nevertheless, phenotypic features should also be considered before those proposals can be formulated.

### Phenotypic Characteristics Endorse the Taxonomic Rearrangements for the Genera *Natrinema* and *Haloterrigena*

For an accurate classification of a taxon, three major premises should be fulfilled: (i) monophyly, (ii) genomic coherence, and (iii) phenotypic coherence (Rosselló-Móra and Amann, 2015). In the previous sections we have examined the two first criteria (phylogenetic/phylogenomic trees and OGRI/synteny), but any formal taxonomic proposal should also be supported by phenotypic characters.

The species *Haloterrigena turkmenica*, *Haloterrigena salifodinae*, and *Haloterrigena salina*, which we propose to be retained as members of the genus *Haloterrigena*, shared a bunch of characteristics (Table 2), such as the coccoid morphology, the red pigmentation, the resistance to lysis in distilled water, the high salt concentration for optimal growth [>15% (w/v) NaCl], the inability to produce gas from nitrate, to form indole and H_2_S, and to hydrolyze starch, gelatin and Tween 80, the ability to use D-glucose, D-mannose and lactose as a sole carbon and energy sources, the presence of phosphatidylglycerol (PG), phosphatidylglycerol phosphate methyl ester (PGP-Me) and mannose-2,6-disulfate (1→2)-glucose glycerol diether (S_2_-DGD-1) as membrane polar lipids, and the lack of phosphatidylglycerol sulfate (PGS). On the other hand, the species *Haloterrigena daqingensis*, which formed a monophyletic cluster with the species of the genus *Natronorubrum*, showed some phenotypic similarities with the species of the latter genus, remarkably, the haloalkaliphilic behavior, the inability to hydrolyze casein and to assimilate D-ribose, D-mannitol and sorbitol, the presence of PG and PGP-Me, and the absence of PGS (Table 2). Finally, the remaining species of *Haloterrigena* together to the species of the genus *Natrinema* and *Halopiger salifodinae* lysed in distilled water, grew optimally in media with 15–29% (w/v) NaCl, utilized acetate but not D-mannitol as the only carbon and energy sources, and possessed PG and PGP-Me as major polar lipids (Table 2). Some differences in the minor polar lipid composition of this *Natrinema*/*Haloterrigena*/*Halopiger salifodinae* group can be observed, in particular, the presence of S_2_-DGD-1 glycolipid in some species and its absence in others, and the lack of PGS in several taxa but not in all of them. Although previous studies have shown that there are differences in the polar lipid composition between the species of the genera *Natrinema* and *Haloterrigena* –with *Natrinema* species harboring PGS but not S_2_-DGD-1 (McGenity et al., 1998; Xin et al., 2000; Xu et al., 2005b; Tapingkae et al., 2008), while *Haloterrigena* representatives containing S_2_-DGD-1 and lacking PGS (Montalvo-Rodríguez et al., 2000; Xu et al., 2005a; Cui et al., 2006b; Roh et al., 2009; Ding et al., 2017)–, we observed that minor polar lipid profiles are not genus-specific. For example, *Natrinema ejinorense* and *Natrinema soli* possessed S_2_-DGD-1 and lacked PGS, and *Natrinema salaciae* contained S_2_-DGD-1 (characteristic profiles of *Haloterrigena* species). On the contrary, *Haloterrigena hispanica* did not hold S_2_-DGD-1 (typical profile of *Natrinema* species). Therefore, those differences in minor polar lipid composition cannot be regarded as phenotypic incoherence within the *Natrinema*/*Haloterrigena*/*Halopiger salifodinae* cluster, whose species should be merged into the single genus *Natrinema*.

**TABLE 2:**
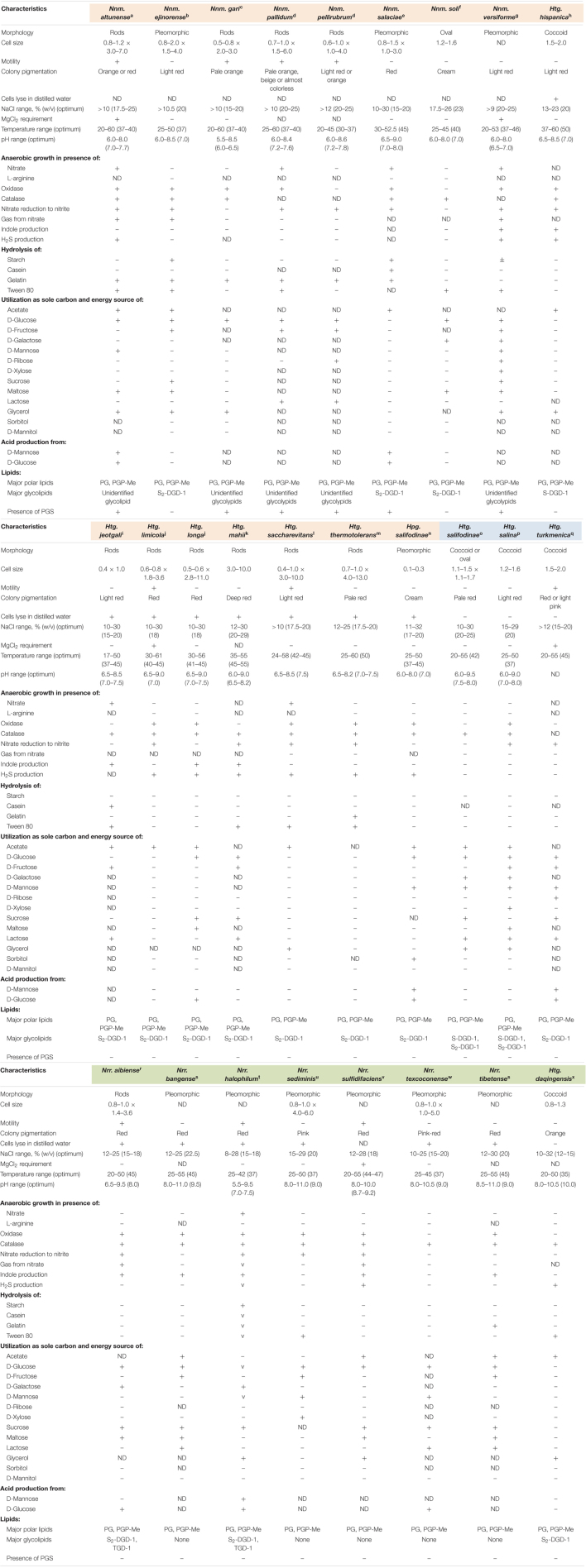
Main comparative phenotypic features among members of the genera *Natrinema* (including the species *Halopiger salifodinae*), *Haloterrigena*, and *Natronorubrum*.

*+, positive; −, negative; ND, not determined; ±, doubtful; v, variable. Species that should be regarded as member of the genera Natrinema, Haloterrigena, or Natronorubrum are marked in light orange, light blue, and light green, respectively.*

*^a^Data from Xu et al. (2005b).*

*^b^Data from Castillo et al. (2006).*

*^c^Data from Tapingkae et al. (2008).*

*^d^Data from McGenity et al. (1998).*

*^e^Data from Albuquerque et al. (2012).*

*^f^Data from Rasooli et al. (2017).*

*^g^Data from Xin et al. (2000).*

*^h^Data from Romano et al. (2007).*

*^i^Data from Roh et al. (2009).*

*^j^Data from Cui et al. (2006b).*

*^k^Data from Ding et al. (2017).*

*^l^Data from Xu et al. (2005a).*

*^m^Data from Montalvo-Rodríguez et al. (2000).*

*^n^Data from Zhang et al. (2013).*

*^o^Data from Chen et al. (2019).*

*^p^Data from Gutiérrez et al. (2008).*

*^q^Data from Zvyagintseva and Tarasov (1987) and Ventosa et al. (1999).*

*^r^Data from Cui et al. (2006a).*

*^s^Data from Xu et al. (1999).*

*^t^Data from Tao et al. (2020).*

*^u^Data from Gutiérrez et al. (2010).*

*^v^Data from Cui et al. (2007).*

*^w^Data from Ruiz-Romero et al. (2013).*

*^x^Data from Wang et al. (2010).*

With respect to genera differentiation, the genuine genus *Haloterrigena* (*Haloterrigena turkmenica*, *Haloterrigena salifodinae*, and *Haloterrigena salina*) can be distinguished from the now expanded genus *Natrinema* (*Natrinema*/*Haloterrigena*/*Halopiger salifodinae* group) by the resistance to cell lysis in distilled water of the former but not of the latter. Likewise, members of the genus *Natronorubrum* (now also including the species *Haloterrigena daqingensis*) are haloalkalophiles, in contrast to their *Haloterrigena* and *Natrinema* counterparts which better thrive at almost neutral pH values (Table 2).

At the species level, phenotypic features can also shed light on uncertain taxa. This is the case of the cluster *Natrinema pellirubrum*/*Haloterrigena jeotgali*/*Haloterrigena thermotolerans* and the cluster *Natrinema ejinorense*/*Haloterrigena longa*, for which OGRI values fell in the fuzzy zone and synteny analysis agreed with the possibility of merging the species within each cluster. A careful inspection of the phenotypic characteristics of *Natrinema pellirubrum*, *Haloterrigena jeotgali*, and *Haloterrigena thermotolerans* demonstrated a similar profile for the two latter, whereas the former showed significant differences as to be considered as a separated species, such as the cell motility, the absence of S_2_-DGD-1 glycolipid and the presence of PGS (Table 2). On the contrary, phenotypic profile for the species *Natrinema ejinorense* and *Haloterrigena longa* was quite similar, with only minor strain-specific differences (Table 2), thus supporting the unification of both taxa into a single species.

### Taxonomic Consequences

After having completed detailed phylogenomic, genomic and phenotypic comparative analyses in the family *Natrialbaceae*, and more specifically in the genera *Natrinema* and *Haloterrigena*, we have demonstrated that the species *Haloterrigena jeotgali* and *Natrinema ejinorense* should be considered as later heterotypic synonyms of the species *Haloterrigena thermotolerans* and *Haloterrigena longa*, respectively, according to Rule 23a of the International Code of Nomenclature of Prokaryotes (Parker et al., 2019). Additionally, the species *Haloterrigena hispanica*, *Haloterrigena limicola*, *Haloterrigena longa*/*Natrinema ejinorense*, *Haloterrigena mahii*, *Haloterrigena saccharevitans*, *Haloterrigena thermotolerans*/*Haloterrigena jeotgali*, and *Halopiger salifodinae* should be transferred to the genus *Natrinema*, as *Natrinema hispanicum*, *Natrinema limicola*, *Natrinema longum*, *Natrinema mahii*, *Natrinema saccharevitans*, *Natrinema thermotolerans*, and *Natrinema salifodinae*, respectively. On the contrary, the species *Haloterrigena turkmenica*, *Haloterrigena salifodinae*, and *Haloterrigena salina* will remain as the only representative species of the genus *Haloterrigena*. Besides, the species *Haloterrigena daqingensis* should be reclassified as a member of the genus *Natronorubrum*, as *Natronorubrum daqingense*.

With regards to non-type or unnamed strains, our study indicates that the strains *Natrinema* sp. J7-1, *Natrinema* sp. J7-2, and *Haloterrigena turkmenica* WANU15 should be renamed as *Natrinema gari* J7-1, *Natrinema gari* J7-2, and *Natronolimnohabitans innermongolicus* WANU15, respectively, although it is worth mentioning that the genome sequence of *Haloterrigena turkmenica* WANU15 has been identified as contaminated in a previous study (Lee et al., 2017). Moreover, the strains *Haloterrigena hispanica* CDM_1 and *Haloterrigena hispanica* CDM_6 should not be longer affiliated to the species *Haloterrigena* (*Natrinema*) *hispanica* and, thus, they should be referred as *Natrinema* sp. CDM_1 and *Natrinema* sp. CDM_6, respectively.

On the basis of these data, we propose the following taxonomic re-arrangements.

**Description of *Natrinema hispanicum* comb. nov.**

*Natrinema hispanicum* (his.pa’ni.cum. L. neut. adj. *hispanicum* of Hispania, from where the organism was originally isolated)

Basonym: *Haloterrigena hispanica* Romano et al., 2007, 1501.

The description is identical to that of *Haloterrigena hispanica* as given previously (Romano et al., 2007) with the following amendments: the G + C content of the type strain genome is 60.7 mol%, its approximate size 4.26 Mb, and its GenBank Assembly accession number is GCA_004217335.1.

The type strain is FP1^T^ (= ATCC BAA-1310^T^ = DSM 18328^T^).

**Description of *Natrinema limicola* comb. nov.**

*Natrinema limicola* (li.mi’co.la. L. masc. n. *limus* mud; L. suff. -*cola* from L. masc. or fem. n. *incola* dweller; N.L. n. *limicola* mud-dweller)

Basonym: *Haloterrigena limicola* Cui et al., 2006b, 1839.

The description is identical to that of *Haloterrigena limicola* as given previously (Cui et al., 2006b) with the following amendments: the G + C content of the type strain genome is 61.8 mol%, its approximate size 3.52 Mb, and its GenBank Assembly accession number is GCA_000337475.1.

The type strain is AX-7^T^ (= CGMCC 1.5333^T^ = JCM 13563^T^).

**Description of *Natrinema longum* comb. nov.**

*Natrinema longum* (lon’gum. L. neut. adj. *longum* long, referring to the production of long rods in liquid medium)

Basonym: *Haloterrigena longa* Cui et al., 2006b, 1838.

The description is identical to that of *Haloterrigena longa* as given previously (Cui et al., 2006b) with the amendments as follows. Cells are rod-shaped or pleomorphic (0.5–2.0 × 1.5–11.0 μm). Aerobic growth occurs at pH 6.0–9.0 and 25–56°C. Optimal NaCl concentration and temperature for growth are 18–20% (w/v) and 37–45°C, respectively. Nitrate reduction to nitrite is variable. Indole and H_2_S formation are variable. Hydrolysis of starch, gelatin and Tween 80 is variable. Assimilation of fructose as carbon and energy sources is variable. Acid production from glucose and sucrose is variable. Phosphatidylglycerol sulfate polar lipid is absent or below detection limit. The DNA G + C content is 61.8–63.9 mol% (genome).

The type strain is ABH32^T^ (= CGMCC 1.5334^T^ = JCM 13562^T^). The G + C content of the type strain genome is 61.8 mol%, its approximate size 3.52 Mb, and its GenBank Assembly accession number is GCA_020105915.1.

*Natrinema ejinorense* EJ-57 (= CECT 7144 = CGMCC 1.6202 = DSM 18194 = JCM 13890) is an additional strain of *Natrinema longa*. The G + C content of this reference strain genome is 63.9 mol%, its approximate size 4.48 Mb, and its GenBank Assembly accession number is GCA_002494345.1.

**Description of *Natrinema mahii* comb. nov.**

*Natrinema mahii* (mah’i.i. N.L. gen. n. *mahii* of Mah, in honor of R.A. Mah at UCLA for his noteworthy research in the areas of archaea isolation and classification, and also for initiating the solar saltern sampling in the original description)

Basonym: *Haloterrigena mahii* Ding et al., 2017, 1337.

The description is identical to that of *Haloterrigena mahii* as given previously (Ding et al., 2017) with the following amendments: the G + C content of the type strain genome is 65.1 mol%, its approximate size 3.79 Mb, and its GenBank Assembly accession number is GCA_000690595.2.

The type strain is H13^T^ (= BCRC 910151^T^ = NBRC 111885^T^).

**Description of *Natrinema saccharevitans* comb. nov.**

*Natrinema saccharevitans* (sac.char.e.vi’tans. L. neut. n. *saccharon*, -*i* a kind of sugar; L. pres. part. *evitans* shunning, avoiding; N.L. part. adj. *saccharevitans* sugar-avoiding, because it uses very few sugars)

Basonym: *Haloterrigena saccharevitans* Xu et al., 2005a, 2541.

The description is identical to that of *Haloterrigena saccharevitans* as given previously (Xu et al., 2005a) with the following amendments: the G + C content of the type strain genome is 65.3 mol%, its approximate size 3.98 Mb, and its GenBank Assembly accession number is GCA_001953745.1.

The type strain is AB14^T^ (= AS 1.3730^T^ = JCM 12889^T^).

**Description of *Natrinema thermotolerans* comb. nov.**

*Natrinema thermotolerans* (ther.mo.to’le.rans. Gr. fem. n. *therme* heat; L. pres. part. *tolerans* tolerating; N.L. part. adj. *thermotolerans* heat-tolerant)

Basonym: *Haloterrigena thermotolerans* Montalvo-Rodríguez et al., 2000, 1070.

The description is identical to that of *Haloterrigena thermotolerans* as given previously (Montalvo-Rodríguez et al., 2000) with the amendments as follows. Cells are 0.4–1.0 × 1.0–13.0 μm. Aerobic growth occurs in the presence of 10–30% (w/v) NaCl, pH 6.5–8.5 and 17–60°C. Optimal NaCl concentration and temperature for growth are 15–20% (w/v) and 37–50°C, respectively. Anaerobic growth in the presence of nitrate is variable. Oxidase activity, reduction of nitrate to nitrite and indole formation are variable. Hydrolysis of casein and gelatin is variable. Assimilation of fructose and lactose as carbon and energy sources is variable. The DNA G + C content is 65.0–65.4 mol% (genome).

The type strain is PR5^T^ (= ATCC 700275^T^ = DSM 11552^T^). The G + C content of the type strain genome is 65.4 mol%, its approximate size 3.90 Mb, and its GenBank Assembly accession number is GCA_000337115.1.

*Haloterrigena jeotgali* A29 (= CECT 7218 = DSM 18794 = JCM 14585 = KCTC 4020) is an additional strain of *Natrinema thermotolerans*. The G + C content of this reference strain genome is 65.0 mol%, its approximate size 4.90 Mb, and its GenBank Assembly accession number is GCA_004799625.1.

**Description of *Natrinema salifodinae* comb. nov.**

*Natrinema salifodinae* (sa.li.fo.di’nae. N.L. gen. fem. n. *salifodinae* of a saltpit, salt mine)

Basonym: *Halopiger salifodinae* Zhang et al., 2013, 3565.

The description is identical to that of *Halopiger salifodinae* as given previously (Zhang et al., 2013) with the following amendments: the G + C content of the type strain genome is 65.4 mol%, its approximate size 4.27 Mb, and its GenBank Assembly accession number is GCA_900110455.1.

The type strain is KCY07-B2^T^ (= CGMCC 1.12284^T^ = DSM 26231^T^ = JCM 18547^T^).

**Description of *Natronorubrum daqingense* comb. nov.**

*Natronorubrum daqingense* (da.qing.en’se. N.L. neut. adj. *daqingense* pertaining to Daqing, north-east China, where the type strain was isolated)

Basonym: *Haloterrigena daqingensis* Wang et al., 2010, 2270.

The description is identical to that of *Haloterrigena daqingensis* as given previously (Wang et al., 2010) with the following amendments: the G + C content of the type strain genome is 61.3–61.4 mol%, its approximate size 3.83–3.84 Mb, and its GenBank Assembly accession numbers are GCA_900156445.1 and GCA_001971705.1.

The type strain is JX313^T^ (= CGMCC 1.8909^T^ = NBRC 105739^T^).

**Emended description of the genus *Natrinema***

*Natrinema* (Na.tri.ne’ma. N.L. n. *natrium* sodium; Gr. neut. n. *nema* a thread; N.L. neut. n. *Natrinema* the sodium thread, referring to the high sodium ion requirement, and the cell shape)

Cells are rods, coccoid or pleomorphic. Cells lyse at low NaCl concentration (<1.0 M). Colonies are red, light orange-red, pale orange-red, or cream pigmented. Chemo-organotroph. Some species are strict aerobes, whereas others show anaerobic growth with nitrate. Catalase positive. Grows on a wide range of substrates, including single and complex carbon sources. Extremely halophilic, requiring at least 9–10% (w/v) NaCl for growth, with optimum at 15–29% (w/v) NaCl. Grows at pH values of 5.5–9.0, with optimum pH at 6.0–8.2. Temperature supporting growth ranges from 17 to 61°C, with optimum at 30–55°C. Possesses C_2__0_C_20_ and C_2__0_C_25_ diether core lipids. The major polar lipids consist of phosphatidylglycerol and phosphatidylglycerol-phosphate-methyl ester, with some species also containing phosphatidylglycerol sulfate. Most species possess the glycolipid S_2_-DGD-1, while some species possess S-DGD-1 or unidentified glycolipids. The DNA G + C content is in the range of 60.7–65.4 mol% (genome). The genus is a member of the family *Natrialbaceae*, order *Natrialbales*, class *Halobacteria*. The recommended three-letter abbreviation is *Nnm*. The type species is *Natrinema pellirubrum*.

**Emended description of the genus *Haloterrigena***

*Haloterrigena* (Ha.lo.ter.ri’ge.na. Gr. n. *hals* halos the sea, salt; L. fem. adj. *terrigena* born from the earth; N.L. fem. n. *Haloterrigena* salt (-requiring) and born from the earth).

Cells are Gram-strain-negative, coccoid, or oval-shaped, and 1.1–2.0 μm in size. Colonies are colored light red or light pink due to the presence of bacterioruberin carotenoids. Cells are non-motile or motile and aerobic. Catalase-positive and oxidase-variable. Extremely halophilic, with growth occurring in media containing 10–30% (w/v) NaCl, with optimum at 15–25% (w/v) NaCl. Cells lyse in distilled water. Species may require or not magnesium to grow. Grows at pH values of 6.0–9.5, with optimum pH at 7.0–8.0. Temperature supporting growth ranges from 20 to 55°C, with optimum at 37–45°C. Some species reduce nitrate to nitrite but they do not form gas from nitrate. Indole formation and H_2_S production are negative. Hydrolysis of starch, gelatin and Tween 80 is negative. Chemo-organotrophic. All species use sugars, some of them with the production of acids. The major polar lipids are C_2__0_C_20_ and C_2__0_C_25_ glycerol diether derivatives of phosphatidylglycerol and phosphatidylglycerol-phosphate-methyl ester as well as the glycolipid S_2_-DGD-1. Some species may also contain the glycolipid S-DGD-1. Phosphatidylglycerol sulfate is absent. The DNA G + C content is between 64.5 and 65.4 mol% (genome). The genus is a member of the family *Natrialbaceae*, order *Natrialbales*, class *Halobacteria*. The recommended three-letter abbreviation is *Htg*. The type species is *Haloterrigena turkmenica*.

**Emended description of the genus *Natronorubrum***

*Natronorubrum* (Na.tro.no.ru’brum. Gr. n. *natron* derived from Arabic *natrun* soda (sodium carbonate); L. neut. adj. *rubrum* red; N.L. neut. n. *Natronorubrum* the red of soda).

Cells are Gram-strain-negative, rods, coccoid or pleomorphic (flat, triangular, square, disc and other polygonal shapes). Colonies are red, pink, or orange pigmented. Cells are non-motile or motile, aerobic or facultative anaerobic. Catalase-positive and oxidase-variable. Extremely halophilic, with growth occurring in media containing 8–32% (w/v) NaCl, with optimum at 12–22.5% (w/v) NaCl. Cells from most species are lysed in distilled water, but others are not. Alkaliphilic or neutrophilic, growing at pH values of 5.5–11.0, with optimum pH at 7.0–10.0. Temperature supporting growth ranges from 20 to 55°C, with optimum at 35–47°C. Chemo-organotrophic. Many substrates are utilized, sometimes with acid production. The major polar lipids are C_2__0_C_20_ and C_2__0_C_25_ derivatives of phosphatidylglycerol and phosphatidylglycerol-phosphate-methyl ester. Phosphatidylglycerol sulfate is absent. Cells may also contain S_2_-DGD-1, TGD-1 and other unidentified glycolipids. The DNA G + C content is in the range of 60.4–63.6 mol% (genome). The genus is a member of the family *Natrialbaceae*, order *Natrialbales*, class *Halobacteria*. The recommended three-letter abbreviation is *Nrr*. The type species is *Natronorubrum bangense*.

## Data Availability Statement

The datasets presented in this study can be found in online repositories. The names of the repository/repositories and accession number(s) can be found in the article/Supplementary Material.

## Author Contributions

RRH, HM, MK, YS, and AV: conceptualization, investigation, writing – review and editing. RRH, HM, and YS: methodology, formal analysis. RRH, HM, and MK: validation. HM, MK, YS, and AV: resources, project administration, and funding acquisition. RRH and HM: data curation, writing – original draft preparation, and visualization. MK, YS, and AV: supervision. All authors have read and agreed to the published version of the manuscript.

## Conflict of Interest

The authors declare that the research was conducted in the absence of any commercial or financial relationships that could be construed as a potential conflict of interest.

## Publisher’s Note

All claims expressed in this article are solely those of the authors and do not necessarily represent those of their affiliated organizations, or those of the publisher, the editors and the reviewers. Any product that may be evaluated in this article, or claim that may be made by its manufacturer, is not guaranteed or endorsed by the publisher.
