# Supplementary material for: Phylogenomics of Haloarchaea: The Controversy of the Genera Natrinema-Haloterrigena
Source: Front Microbiol. 2021 Oct 7;12:740909. doi: 10.3389/fmicb.2021.740909 (PMC8530250; doi:10.3389/fmicb.2021.740909)
Supplement: Supplementary file 1 [file Data_Sheet_1.pdf]

Supplementary Material

Phylogenomics of Haloarchaea: The Controversy of the Genera *Natrinema-Haloterrigena*

Rafael R. de la Haba[1], Hiroaki Minegishi[2], Masahiro Kamekura[3], Yasuhiro Shimane[4], Antonio Ventosa[1,*]

[1]Department of Microbiology and Parasitology, Faculty of Pharmacy, University of Sevilla, Sevilla, Spain
[2]Department of Applied Chemistry, Faculty of Science and Engineering, Toyo University, Kawagoe, Saitama, Japan
[3]Halophiles Research Institute, Noda, Chiba, Japan
[4]Japan Agency for Marine-Earth Science and Technology, Yokosuka, Kanagawa, Japan

*** Correspondence:**
Antonio Ventosa
ventosa@us.es

1 Supplementary Figures

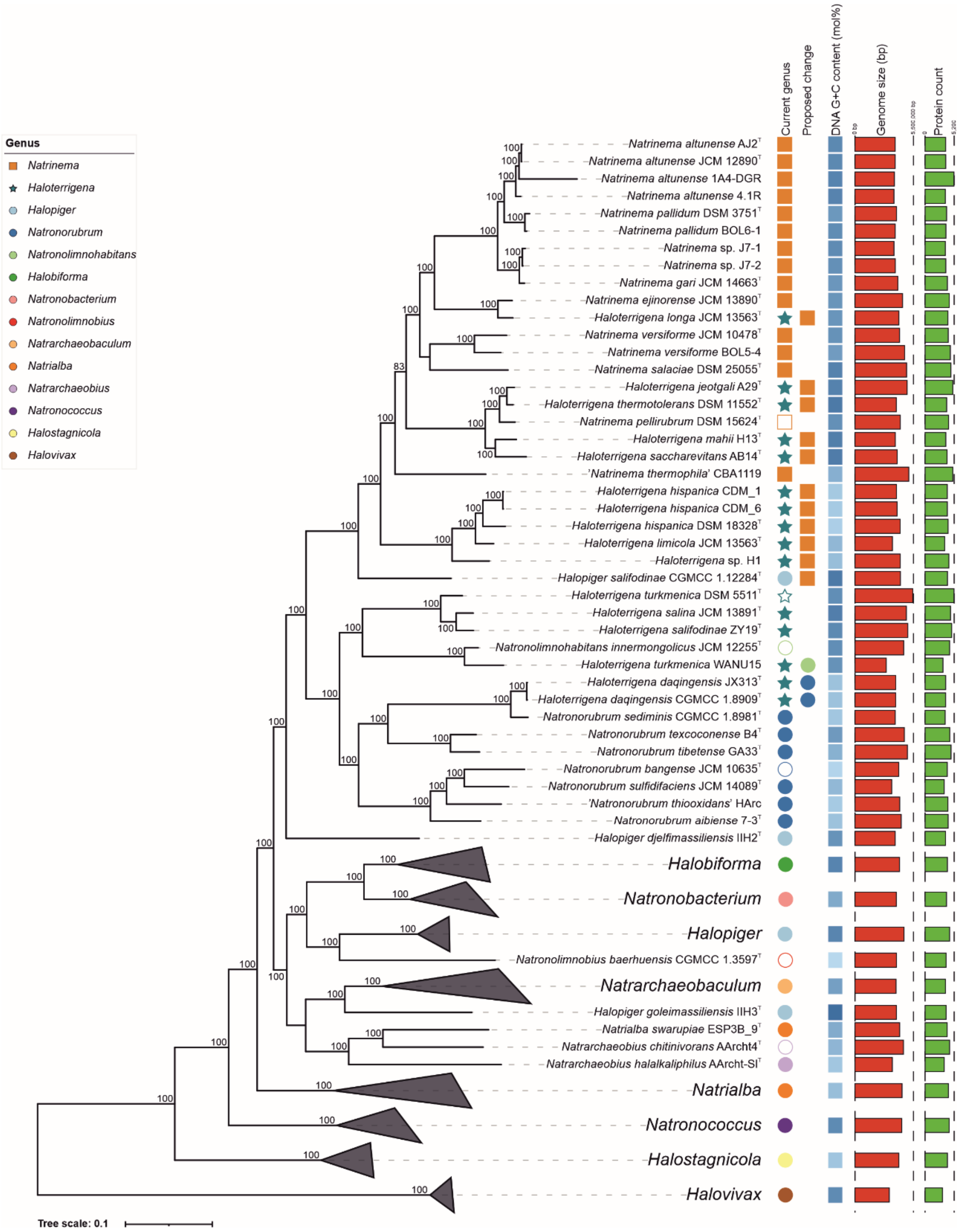

Supplementary Figure 1. Approximate maximum-likelihood phylogenomic tree based on the concatenation of the translated sequence of the 525 single-copy genes shared by all the members of the genera *Natrinema* and *Haloterrigena* and related taxa of the family *Natrialbaceae* under study. Bootstrap values ≥ 70% (based on Shimodaira-Hasegawa-like local support) are shown above the branches. Bar, 0.1 changes per nucleotide position. Empty symbols indicate the type species of the corresponding genus.

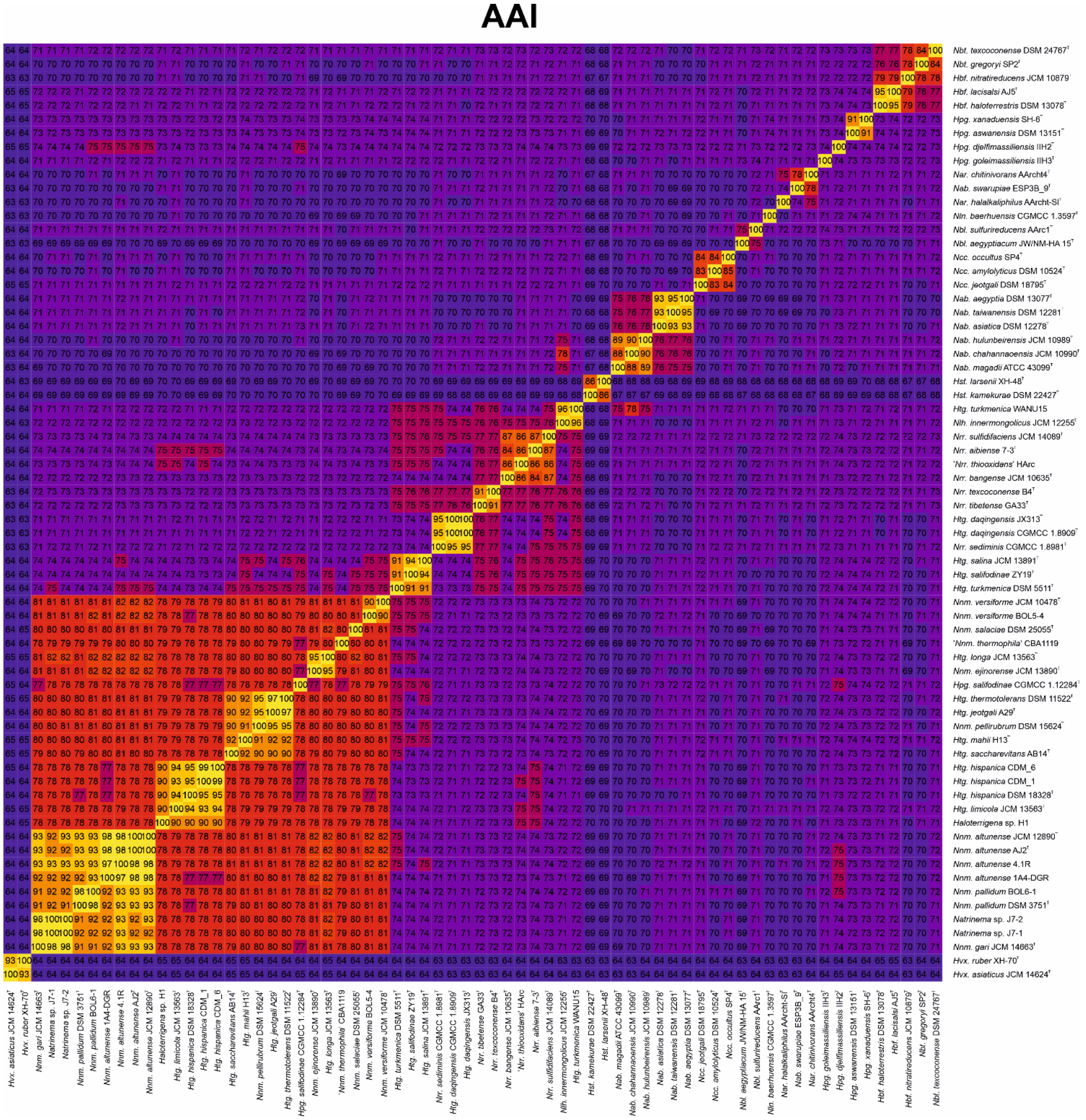

Supplementary Figure 2. Heatmap of AAI relatedness among all the members of the genera *Natrinema* and *Haloterrigen*a under study and representatives of the other genera of the family *Natrialbaceae*.

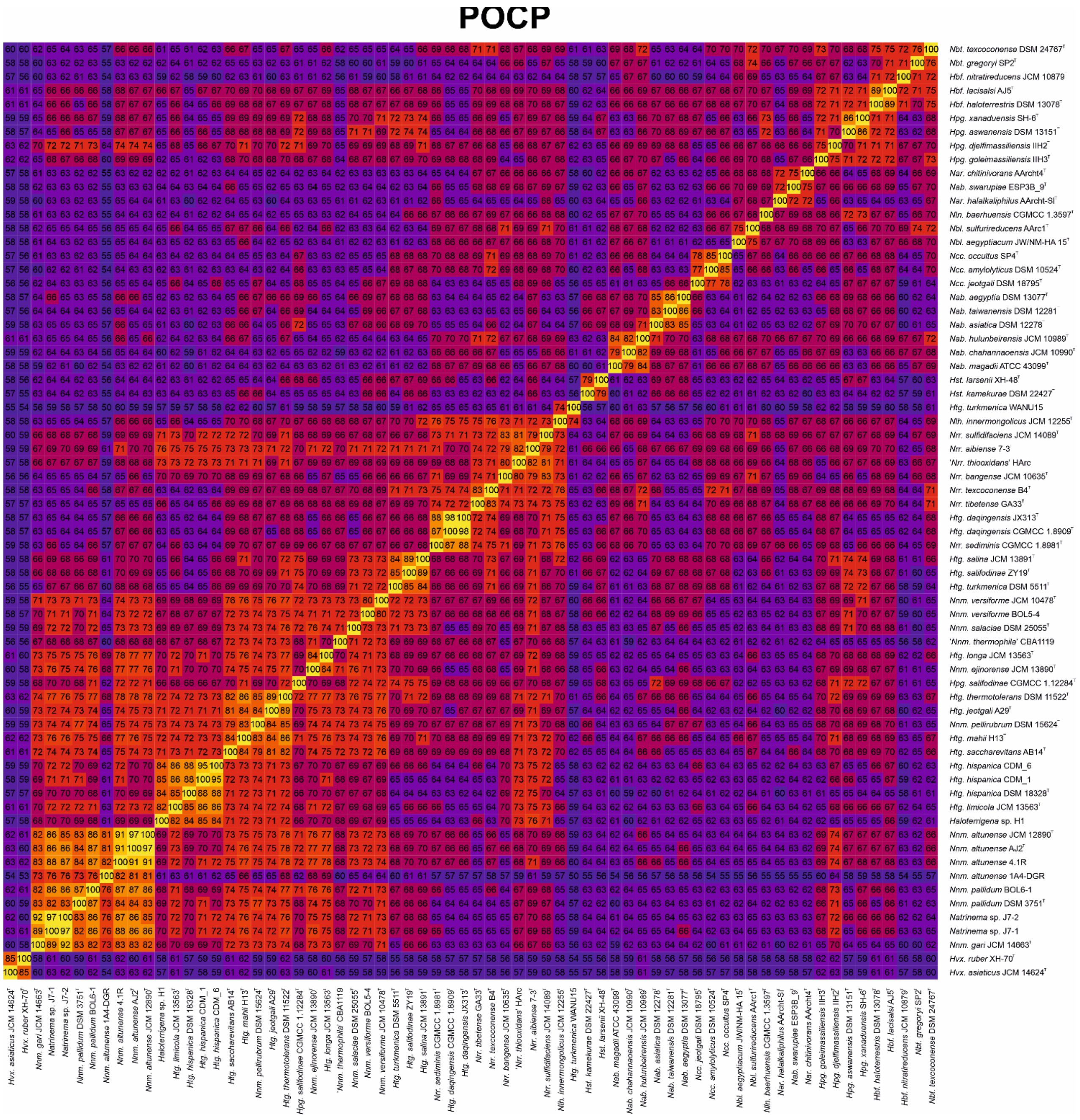

Supplementary Figure 3. Heatmap of POCP relatedness among members of the genera *Natrinema* and *Haloterrigen*a and representatives of the other genera of the family *Natrialbaceae*.

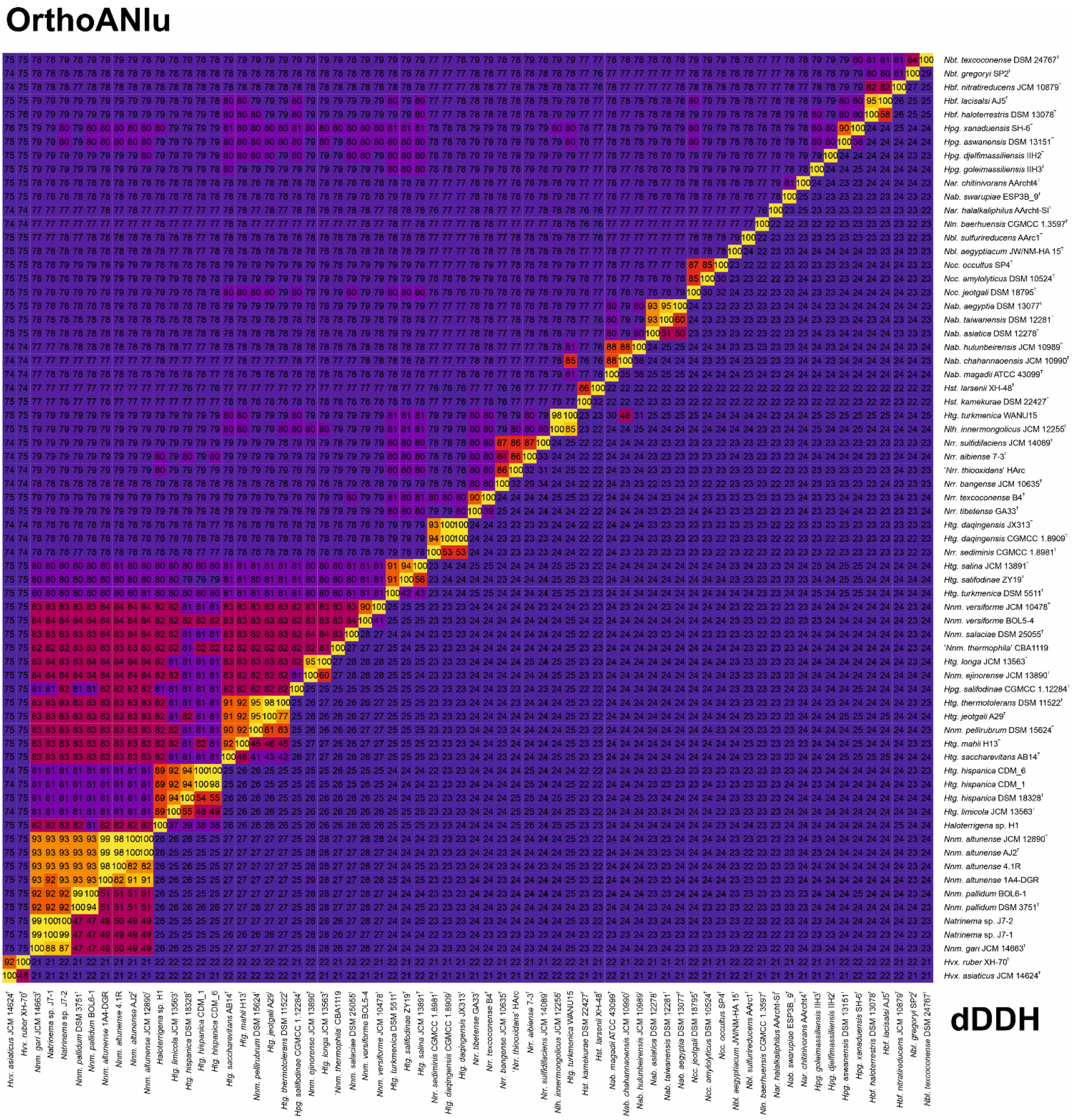

Supplementary Figure 4. Heatmap of OrthoANIu (upper triangle) and dDDH (lower triangle) relatedness among all the members of the genera *Natrinema* and *Haloterrigen*a under study and representatives of the other genera of the family *Natrialbaceae*.
